# Letter to the editor: dynamic ctDNA monitoring in adjuvant therapy and recurrence for colorectal liver metastases: a prospective study

**DOI:** 10.1097/JS9.0000000000002843

**Published:** 2025-07-07

**Authors:** Yujun Yu, Yongjun Jin

**Affiliations:** Department of Colorectal Surgery, Hangzhou Red Cross Hospital, Hangzhou, Zhejiang, China

*Dear Editor,*

We read with great interest the recently published article titled *“The Value of Postoperative Serial ctDNA Monitoring in Guiding Adjuvant Therapy and Predicting Recurrence in Patients with Colorectal Liver Metastases”* in your journal^[1]^. This prospective study explores the potential role of circulating tumor DNA (ctDNA) in evaluating recurrence risk and guiding adjuvant treatment decisions following curative resection of colorectal liver metastases. By employing the J25 tumor-informed panel for longitudinal ctDNA monitoring, the authors rigorously analyzed its association with adjuvant chemotherapy (ACT) response, recurrence timing, radiological progression, and survival outcomes. The study is well-designed and reflects the forefront application of ctDNA in precision oncology. We sincerely commend the authors for their meticulous work. While the findings are promising, we believe several points merit further discussion to enhance the scientific rigor and clinical utility of the study:

First, this is a single-center, non-randomized prospective study. Although it provides valuable real-world data, the limited sample size and potential biases in patient selection and testing intervals may affect the generalizability of the conclusions. Notably, there is still no universally accepted threshold for defining ctDNA positivity^[2]^. Although the panel design was based on archived tissue samples from the study center and validated in prior work, a clearer standard linking ctDNA quantification to clinical events (e.g., recurrence timing, overall survival) would improve the interpretability and practical application of ctDNA results. Additionally, ctDNA status may not necessarily be a binary variable. A multi-class categorization, such as low, intermediate, and high ctDNA levels, could offer a more nuanced understanding. Incorporating machine learning models into the analysis could enable more refined risk stratification and prediction of recurrence or prognosis based on ctDNA dynamics^[3]^.

The study highlights the prognostic importance of ctDNA dynamics, particularly at the 1-month postoperative mark. However, from a clinical perspective, the high cost of ctDNA testing raises the question of whether routine testing every three months is truly necessary. Future research should aim to identify an optimal balance between testing frequency, duration, and clinical benefit. Moreover, integrating ctDNA analysis with other biomarkers may provide a more comprehensive assessment of disease status and enhance both accessibility and cost-effectiveness in clinical practice^[4]^.

Furthermore, while the study demonstrates a strong correlation between ctDNA positivity and early recurrence, the analysis of the time to recurrence and organ-specific patterns about ctDNA levels remains limited. We recommend further exploration using multivariable regression models to assess the relationship between ctDNA clearance and different recurrence types (such as intrahepatic recurrence vs. distant metastasis), which could inform more personalized follow-up strategies.

In summary, this study provides valuable evidence supporting the clinical utility of ctDNA in postoperative management of CRLM and advances the application of liquid biopsy in precision oncology. We sincerely thank the authors for their important contribution and look forward to future studies with larger cohorts, multicenter participation, and randomized designs to further validate and refine the clinical standards for ctDNA implementation.

## Data Availability

Not applicable.
